# The Effect of Active Transport, Transport Systems, and Urban Design on Population Health

**DOI:** 10.1155/2013/457159

**Published:** 2013-11-11

**Authors:** Li Ming Wen, Chris Rissel, Hua Fu

**Affiliations:** ^1^Sydney School of Public Health, University of Sydney, Australia; ^2^Health Promotion Service, Sydney Local Health District, NSW, Australia; ^3^School of Public Health, Fudan University, China

Without doubt, active transport (working, cycling, and the use of public transport), transport systems, and urban design significantly impact population health. A 2013 systematic review of the relationships between active transport and health outcomes found that active transport was significantly associated with improved cardiovascular health and lower body weight. However, the strength of the evidence varied from weak (mental health and cancer), moderate (body weight), to strong (cardiovascular health). The evidence was limited by lack of comparability of study outcomes, weak study designs, small sample sizes, and lack of experimental studies [1]. 

Although the links between active transport and population health are still emerging, the evidence linking active transport directly to health outcomes has not been examined extensively and the relationship between active transport and health still remains unclear. More research is needed to better understand the important correlates related to active transport and its contributors to population health, and develop effective strategies to promote active transport. With this in mind we selected the theme for this special issue. We wish to highly commend the authors for their well written papers exploring a range of issues related to promoting active transport, which include health benefits associated with it and potential strategies to promote active transport as well as challenges in measuring the effectiveness of promoting active transport. 

The paper by M. Bopp et al. examined the association between health-related factors and mode of travel to the workplace. The study used a convenience sample of employed adults who completed an online survey regarding demographics, health-related factors, and the number of times per week of walking, biking, driving, and using public transit to work. Logistic regression was used to predict the likelihood of each mode of transport and meeting physical activity (PA). The study found that about 10 percent of respondents met PA recommendations based on the use of active transport to work alone, which has an important implication for promoting physical activity through active transport. 

The paper by R. Zhou et al. examined the perceived neighborhood environment characteristics associated with PA in urban areas in China. Cross-cultural comparisons of how the environment can influence behaviour warrant more attention. In this study, adult cross-sectional data was collected using accelerometers, the International Physical Activity Questionnaire (IPAQ), and Neighborhood Environment Walkability Scale-Abbreviated (NEWS-A) survey. Consistent with western countries, residents from downtown areas had higher levels of transportation PA and leisure-time PA than respondents living in the suburbs. Residential density was positively associated with recreational and leisure-based PA, and moderate-vigorous PA was found to be negatively associated with traffic safety. 

An interesting paper by S. R. Lu et al., however, found that, contrary to expectations, there was no benefit to population health from active transport. They conducted a population-based cross-sectional study in Jiangsu, China. 8400 community residents aged 18 or above were interviewed following a multistage random sampling method. Face-to-face questionnaire survey data, anthropometric measurements, and biochemical data from blood tests were collected. Results showed an inverse correlation between active transport and some health outcomes, including cholesterol disorder, risk of diabetes, and risk of obesity.

Another paper focused on cycling was the paper by N. T. R. Romanow et al., which used a case-control study design to examine environmental risk factors for bicycling injuries. They combined data on bicyclist injuries collected by interviews in the emergency department with street-level environmental audits of injury locations, capturing path, roadway, safety, land use, and aesthetic characteristics. They focused on more severe injuries—being struck by a motor vehicle or cyclists that were hospitalized. Adjusted logistic regression analyses found that the factors contributing to motor vehicle events included greater traffic volume intersections, retail establishments, and path obstructions. Protective factors included locations where the road was in good condition and where there was high surveillance from surrounding buildings. Safety issues are an ongoing concern in the promotion of cycling.

C. Rissel et al. conducted two pilot studies on the effect of bicycling on balance and leg strength among older adults. The preliminary results showed that participants who had cycled in the last month performed significantly better on balance measures of decision time and response time in Study 1; and cycling at least one hour a week was associated with significant improvements in balance (decision time and response time) and timed single leg standing from Study 2.

In addition, the paper by T. Xia et al. reviewed the evidence of links between vehicle emissions and air quality, as well as the health and economic benefits from alternative transport use and methodological issues relating to the modeling of these cobenefits. The review concluded that current analyses of transport mitigation strategies in terms of health and economic aspects are still at an early stage. Most of the previous research regarding the transport sector has only focused on one of those possible benefits and has rarely quantified the overall cobenefits of alternative transportation planning. Additionally, most of the current co-benefit studies are more interested in the long-term effect of alternative transportation, while the short-term effect has not been considered adequately. Some other benefits, such as social benefits from alternative transport are also valuable for further investigation.

The paper by N. Petrunoff et al. assessed the reliability and validity of survey questions used to measure workplace travel behavior. Sixty-five respondents completed a travel diary for a week, wore an accelerometer over the same period, and twice completed an online travel survey (21 days apart). They confirmed that the survey question “How did you travel to work this week?” with daily response options over seven days is reliable and agrees well with a travel diary.

D. Cooley and S. Pedersen conducted a pilot study to test the feasibility of a workplace e-health intervention based on a passive approach to increase nonpurposeful movement as a means of reducing sitting time. The study that was trialed in a professional workplace with forty-six participants had some promising results, but there is a need for further investigation. S. Crawford and J. Garrard presented a mixed method study describing a comprehensive impact process evaluation of the Ride2School program in metropolitan and regional areas in Victoria, Australia. The paper highlights the benefits of undertaking both qualitative and quantitative methods in evaluating active transport to school programs that enables both measurement and understanding of program impacts.

Finally, the paper by D. Fuller and P. Morency called for a population approach to transportation planning: reducing exposure to motor-vehicles. This paper provided an overview of Geoffrey Rose's strategy of preventive medicine applied to road traffic fatalities and concluded that interventions should address the large number of people exposed to the fundamental causes of diseases. Public health and transportation research must critically appraise their practice and engage in informed dialogue with the objective of improving mobility and productivity while simultaneously reducing the public health burden of road deaths and injuries.

*Li Ming Wen* *Li Ming Wen* *Chris Rissel* *Chris Rissel* *Hua Fu* *Hua Fu*
